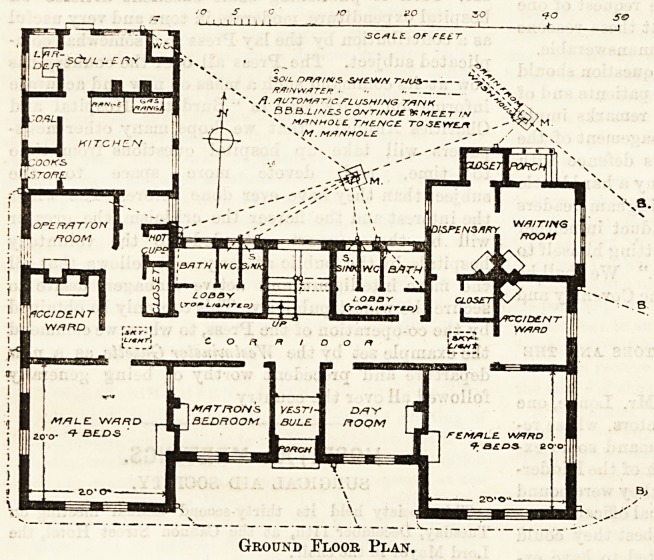# Falmouth Cottage Hospital

**Published:** 1894-12-15

**Authors:** 


### The Institutional Workshop.

### HOSPITAL CONSTRUCTION.

### FALMOUTH COTTAGE HOSPITAL.

So many very excellent cottage hospitals have been built of late years, and so much publicity has been given to plans of all kinds of hospitals, that we should have thought it was an easy task to plan a hospital for ten beds without violating the first rules of hospital sanitation. The present is, however, one of those cases which shows how difficult it appears to be to some architects to learn from what has gone before. Here is an example of a one-storey building with the corridor so badly planned that top lighting has to be resorted to to get sufficient light; with the water-

Ground Floor Plan.

closets separated only by a top lighted lobby from the main corridor into which the wards open; with bathrooms so small and approached in such a way, that to carry a helpless patient in and bath him would be an impossibility; with the kitchen offices and the operation room in the closest proximity, and with the out-patient room opening close on to two wards. The operation room, which is very small, has its window placed right up in one corner and close to the fireplace, so that it would be impossible to put the table opposite the window, and at the same time have free space on both sides of it.

We should recommend the architect of this hospital to apply himself to the perusal of "Burdett's Cottage Hospitals," a book which, though published some fourteen years ago, will supply him with sufficient information to enable him to steer clear of the serious mistakes in the plans we have been criticising.

## Figures and Tables

**Figure f1:**